# The Effects of Botulinum Toxin Injections on Spasticity and Motor Performance in Chronic Stroke with Spastic Hemiplegia

**DOI:** 10.3390/toxins12080492

**Published:** 2020-07-31

**Authors:** Yen-Ting Chen, Chuan Zhang, Yang Liu, Elaine Magat, Monica Verduzco-Gutierrez, Gerard E. Francisco, Ping Zhou, Yingchun Zhang, Sheng Li

**Affiliations:** 1 Department of Physical Medicine and Rehabilitation, University of Texas Health Science Center at Houston, Houston, TX 77030, USA; cheny@nsuok.edu (Y.-T.C.); elaine.m.magat@uth.tmc.edu (E.M.); gutierrezm19@uthscsa.edu (M.V.-G.); gerard.e.francisco@uth.tmc.edu (G.E.F.); 2 TIRR Memorial Hermann Hospital, Houston, TX 77030, USA; 3 Department of Health and Kinesiology, Northeastern State University, Broken Arrow, OK 74014, USA; 4 Department of Biomedical Engineering, University of Houston, Houston, TX 77204, USA; czhang19@uh.edu (C.Z.); yliu77@uh.edu (Y.L.); yzhang94@Central.UH.EDU (Y.Z.); 5 Department of Rehabilitation Medicine, University of Texas Health Science Center at San Antonio, San Antonio, TX 78229, USA; 6 Guangdong Provincial Work Injury Rehabilitation Center, Guangzhou 510000, China; dr.ping.zhou@outlook.com

**Keywords:** botulinum toxin, spasticity, stroke, motor control, force variability, motor performance

## Abstract

Spastic muscles are weak muscles. It is known that muscle weakness is linked to poor motor performance. Botulinum neurotoxin (BoNT) injections are considered as the first-line treatment for focal spasticity. The purpose of this study was to quantitatively investigate the effects of BoNT injections on force control of spastic biceps brachii muscles in stroke survivors. Ten stroke survivors with spastic hemiplegia (51.7 ± 11.5 yrs; 5 men) who received 100 units of incobotulinumtoxinA or onabotulinumtoxinA to the biceps brachii muscles participated in this study. Spasticity assessment (Modified Ashworth Scale (MAS) and reflex torque) and muscle strength of elbow flexors, as well as motor performance assessment (force variability of submaximal elbow flexion) were performed within one week before (pre-injection) and 3~4 weeks (3-wk) after BoNT injections. As expected, BoNT injections reduced the MAS score and reflex torque, and elbow flexor strength on the spastic paretic side. However, motor performance remained within similar level before and after injections. There was no change in muscle strength or motor performance on the contralateral arm after BoNT injections. The results of this study provide evidence that BoNT injections can reduce spasticity and muscle strength, while motor performance of the weakened spastic muscle remains unchanged.

## 1. Introduction

Spasticity and weakness (i.e., spastic hemiparesis) are primary motor impairments and impose significant challenges for patient care. Directly from damage to motor cortex and its descending pathways and lack of central activation [1], weakness is the primary contributor of motor impairments and disabilities [2]. Spasticity is estimated to be present in about 20–40% of all stroke survivors [3], but in up to 97% of those with moderate to severe motor impairment [4]. Clinically, post-stroke spasticity is easily recognized as a phenomenon of velocity-dependent increase in tonic stretch reflexes (‘muscle tone’) with exaggerated tendon jerks, resulting from hyperexcitability of the stretch reflex [5]. Such phenomenon of increased resistance is commonly assessed by passive stretch in clinical practice using clinical scales, such as Modified Ashworth Scale (MAS) and Tardieu scale [6]. However, it is important to point out that not all hypertonia (increased muscle resistance) is stretch reflex mediated spasticity [7]. Quantification of resistance torques in response to motorized stretch in laboratory settings is often able to differentiate neural (i.e., stretch reflex) and non-neural components of hypertonia [8,9,10,11]. Spasticity can cause problems directly, such as abnormal joint position and posture, pain, and hygiene difficulties. It also interacts with and amplifies the effects of other impairments, such as weakness and disordered motor control and impaired coordination, thus contributing to limitations in activity and participation [12]. Stroke survivors often have difficulty to initiate and to terminate a grip on the spastic paretic side [13,14]. During constant force production, it is difficult for them to maintain a steady force on the impaired side as compared to the contralateral side [15,16,17]. The weaker the spastic muscle, the greater the force variability observed [15]. Taken together, spasticity and hemiparesis impose a great challenge on stroke survivors’ performance of activities of daily living, thus quality of life [3].

Chemodenervation with botulinum toxin (BoNT) is the preferred treatment option for focal spasticity management after a stroke, among a spectrum of treatment options [18]. The clinical effects of BoNT do not manifest until several days following an injection, and reach its peak effect around 3~4 weeks. The clinical effects last about three months [19,20]. Usually, patients require repeated BoNT injections every 3~4 months [18,21]. Over past 30 years, accumulated evidence has established the effectiveness of BoNT therapy [22,23,24,25,26,27,28,29,30,31,32,33]. In a recent meta-analysis study of 40 clinical trials [34], Andringa et al. found that there was robust evidence of BoNT on reducing spasticity, as measured with the (Modified) Ashworth Score, and improving self-care ability for the impaired side. BoNT significantly reduced ‘involuntary movements’, However, no evidence was found for functional improvement in ‘arm and hand use’ after BoNT therapy.

Botulinum toxin exerts its effect through inhibition of acetylcholine release at the neuromuscular junction via a complex pross [35,36,37]. Since acetylcholine triggers muscle contraction, when BoNT is precisely injected into a muscle, a locally-confined neuromuscular block develops, leading to paresis of the targeted spastic muscle, thus spasticity reduction. Therefore, weakening of spastic muscles often accompanies the therapeutic effects of spasticity reduction after BoNT injections [38]. Spastic muscles are usually weak. Further weakening of spastic muscles could worsen their motor performance. As mentioned above, the ability of maintaining a steady force output depends on the strength of spastic muscles [15]. Weakening after BoNT injections could have functional consequences as well. For example, BoNT injections to spastic quadriceps are able to reduce spasticity, but the resultant weakness may make weight bearing on the impaired side difficult. This BoNT-related muscle weakness is not well studied, but may contribute to the observation that BoNT therapy does not lead to functional improvement [34]. However, there are unusual cases when the outcome of BoNT injections surpasses this expectation and results in increase in functional abilities in chronic stroke survivors [13,39,40].

The aim of this study was to investigate spasticity reduction and weakness of spastic muscles after BoNT injections and their effects on motor performance. A convenient cohort of stroke survivors with spastic hemiplegia were recruited from our spasticity management clinic. Each subject received 100 units of onabotunilum toxin A or incobotulinum toxin A to spastic biceps brachii muscles. The effect on elbow flexor spasticity was assessed clinically using MAS, and was quantified by reflex torque and non-reflex torque using a biomechanical assessment system. Muscle strength and variability of steady force output were measured during isometric elbow flexion tasks from both spastic paretic and contralateral sides before and after BoNT injections. We hypothesized that BoNT injections would be able to reduce elbow flexor spasticity, and to cause weakness of elbow flexors on the impaired side. Furthermore, muscle performance, as measured by force variability during steady force output, would be further impaired.

## 2. Results

### 2.1. Elbow Flexor Spasticity

As listed in Table 1, elbow flexor spasticity was decreased in 8 of 10 subjects when assessed during the 3-wks visit. Statistically, BoNT injections significantly reduced the MAS score of elbow flexors (*p* < 0.05). As part of exclusion criteria, patients who received injections to other elbow flexors (brachialis and brachioradialis muscles) were excluded. All subjects had injections to other spastic muscles, e.g., finger flexors. Assessment of these spastic muscles were not recorded as they were not the focus of this study. 

### 2.2. Maximum Voluntary Contraction (MVC) Tasks

**Figure 1:**
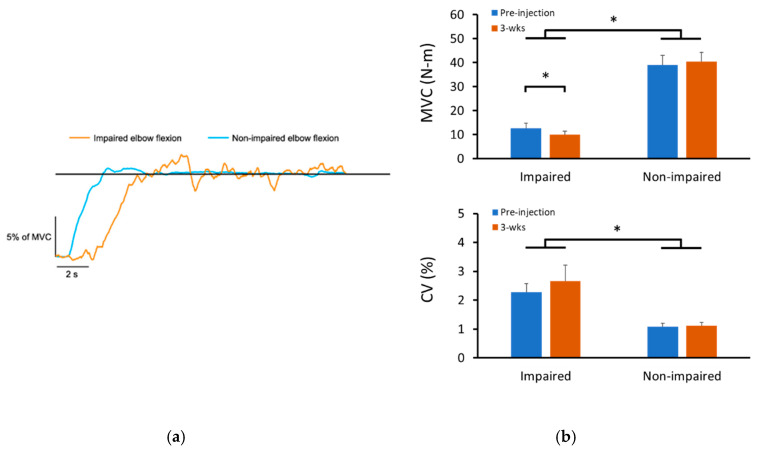
MVC and force control performance. (**a**) Representative trials of force control performance from impaired and non-impaired elbow flexion of one subject; the subject was instructed to keep force traces as close to the target (the horizontal line) as possible. (**b**) MVC force (top) and force control performance averaged over three force levels (bottom) of both impaired and non-impaired elbow flexion before and after BoNT injections. MVC: maximal voluntary contraction; CV: coefficient of variation of force production. * indicates statistically significant difference (*p* < 0.05). BoNT = botulinum toxin.

On average, BoNT injections significantly reduced force production. MVC was 9.98 ± 4.64 N-m during the 3-wks follow up visit compared to 12.73 ± 6.37 N-m during the pre-injection visit (*p* < 0.01, Figure 1b; top). The decrease was about 18.5 ± 18.5%. As expected, the MVC of the non-impaired elbow flexion didn’t change significantly after injections (pre-injection: 38.93 ± 12.88 N-m; 3-wks: 40.47 ± 11.52 N-m).

### 2.3. Force Control Performance

Representative force control trials during 10% of MVC contraction of both impaired and non-impaired side are demonstrated in Figure 1a. There was no significant BoNT injection effect on force control performance. There was no main effect of TIME in 3-way ANOVA tests (*p* = 0.27, partial η^2^ = 0.15) However, there was a significant main effect of LIMBS (impaired and non-impaired) on the coefficient of variation (CV) of force (*p* < 0.05, partial η^2^ = 0.54). Furthermore, there was a significant interaction between LIMBS and FORCELEVEL (10%, 30%, and 50% of MVC; *p* < 0.05, partial η^2^ = 0.44). Post-hoc analysis revealed that CV of force was significantly larger in the impaired side compared to the non-impaired side during 10% of MVC task (impaired: 4.07 ± 1.15%; non-impaired: 1.29 ± 0.29%; *p* < 0.05, partial η^2^ = 0.54) and 30% of MVC task (impaired: 1.89 ± 0.44%; non-impaired: 0.89 ± 0.09%; *p* < 0.05, partial η^2^ = 0.47). All other interactions were not statistically significant (all *p* > 0.05). Given the expected no change in force control performance in the non-impaired side, separate two-way ANOVA tests were performed for each limb with factors of TIME and FORCELEVEL Comparable to the findings in the three-way ANOVA test, CV of force was similar before and after injections in both impaired (pre-injection: 2.28 ± 1.22%%; 3-wks: 2.66 ± 2.26%; *p* > 0.05, partial η^2^ = 0.08) and non-impaired (pre-injection: 1.08 ± 0.43%; 3-wks: 1.13 ± 0.54%; *p* > 0.05, partial η^2^ = 0.18) elbow.

### 2.4. Reflex Torque

**Figure 2:**
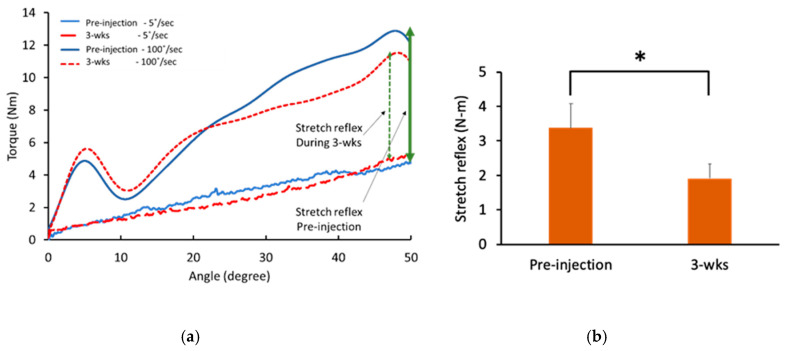
Reflex torque decreased after BoNT injections. (**a**) Representative trials during two different speeds of stretch pre-injection and 3-wks after BoNT injections; (**b**) BoNT injections significantly reduced reflex torque. * indicates statistically significant difference (*p* < 0.05).

BoNT injections significantly decreased the total torque and the reflex torque. Specifically, the total torque significantly reduced at three weeks after BoNT injections (5.47 ± 2.37 N-m) compared to pre-injection (7.07 ± 3.00 N-m, *p* < 0.01). Furthermore, the reflex torque significantly reduced at three weeks after BoNT injections (1.91 ± 1.35 N-m) compared to pre-injection (3.37 ± 2.25 N-m, *p* < 0.01; Figure 2). On average, there was a decrease of 40.8 ± 27.7%. As expected, the baseline torque was not affected by BoNT injections (pre-injection: 3.70 ± 1.30 N-m; 3-wks: 3.56 ± 1.32 N-m; *p* = 0.5). Correlations between reflex torque and muscle strength (MVC) and between reflex torque and force variability (CV) were performed before and after BoNT injections. No significant correlations were found.

## 3. Discussion

In this study, 10 stroke subjects with chronic spastic hemiplegia received a total of 100 units of botulinum toxin A (ona- or inco-botulinum toxin A) to their biceps brachii muscles as their routine treatment of spasticity. Our results confirmed that BoNT injections reduced elbow flexor spasticity in 8 out of 10 subjects and weakened strength of elbow flexors on the impaired side in all subjects. Our findings also confirmed that force variability during isometric elbow flexion was greater on the impaired side than on the contralateral side. However, to our surprise, there was no change in force variability after BoNT injections to the spastic biceps brachii muscles, i.e., the weakened elbow flexors maintained the same level of steady force output. This unexpected novel finding of unchanged motor performance after BoNT injections disputed our hypothesis, however it sheds new light on the application of BoNT for management of spasticity in spastic muscles with residual voluntary control.

### 3.1. Effects of BoNT on and Quantification of Spasticity

BoNT injections were able to successfully reduce elbow flexor spasticity. The Modified Ashworth Scale (MAS) scores of elbow flexors were decreased in 8 of 10 subjects, while MAS scores remained the same for the remaining two subjects. This could be due to a number of reasons. Only biceps brachii muscles were injected in this study, while other elbow flexor muscles (brachialis, and brachioradialis) were not injected based on clinical judgement of our experienced physicians who collectively have performed more than 10,000 BoNT injections. However, these muscles may be mildly spastic and contribute to resistance to passive stretch of the elbow joint. The dose of BoNT may be not adequate for these two subjects, even with the same MAS scores as compared to others. MAS scores may be confounded by co-existing contracture of spastic muscles [41]. Alternatively, it may be the case that MAS is not sensitive to quantify the effect of BoNT injections. In a previous study [42], Pandyan et al. reported that 8 out of 14 stroke subjects did not show MAS improvement after onabotulinum toxin A injections to biceps brachii muscles, even though the BoNT effect was reflected in electromyographic activities. It is likely that the clinical scales, such as MAS, lacks the sensitivity to quantify the abnormal tone with common definitions of stretch-reflex mediated spasticity [43]. In this study, we used an established biomechanical assessment method to quantify and differentiate reflex and non-reflex components of spastic hypertonia [10,11,44]. The results demonstrated significant decrease in the reflex component of spasticity, while the non-reflex muscular component remained unchanged after BoNT injections.

### 3.2. Motor Performance after BoNT

The ability to maintain a steady force output is an essential component of motor control and functional task performance, such as holding a cup of coffee. Findings in this study and results from previous studies [15,16,17] all demonstrate that force variability was greater on the spastic paretic side than on the contralateral side. In our previous study, we further found that there was a significant negative correlation between force variability and weakness of spastic muscles, i.e., the weaker the spastic muscle, the greater force variability is observed [15]. The primary explanation was that the magnitude of force variability was dependent primarily on the number of motor units innervating a target muscle, relating to the muscle strength in healthy subjects [45]. A different interpretation is that force variability could be viewed as “motor noise” of isometric force production. A stronger muscle with a larger number of motor units would have less “motor noise” during voluntary contraction. As mentioned in the introduction, BoNT injections cause partial neuromuscular block in the spastic muscle, thus less motor units, and in turn, muscle weakness and greater “noise” or force variability. Therefore, in addition to spasticity reduction and muscle weakness, greater force variability is expected after BoNT injections. However, our finding of unchanged force variability after BoNT injections suggests that there is another factor of equal importance in spastic muscles to counter balance the effect of neuromuscular block and muscle weakness by BoNT. 

Spasticity reduction after BoNT could be the factor contributing to unchanged force variability during voluntary muscle contraction. Previous studies have demonstrated spontaneous motor discharges in resting spastic muscles of stroke survivors [46,47,48]. Furthermore, the firing rate of these spontaneously firing motor units increased in concert with the level of voluntary contraction [47], and maintained after termination of force production [15]. Spontaneous discharges from these motor units are not under voluntary control and could be viewed as “noises”. These noises are likely to contribute to force control of spastic muscles. Neuromuscular block of these motor units by BoNT also reduce the number of these spontaneously firing motor units, thus reducing the noise from these motor units during voluntary contraction of spastic flexors. In other words, the benefits of suppression of involuntary firing from spontaneous motor units could balance the loss of “normal” motor units after BoNT-related neuromuscular block. Our results showed that stretch reflex torque were not significantly correlated with either muscle strength (MVC) or force variability (CV). These results support the notion that mechanisms mediating muscle strength, and its voluntary control and spasticity, are mediated by two different mechanisms [49]. Spasticity is attributed to unopposed descending excitatory input to spinal reflex circuits from brainstem, and is related to disordered motor control [49]. In a recent study, it was found that reflex torque was correlated with spontaneous activity of spastic muscles [11]. Therefore, reduction of reflex torque after BoNT injections could indicate suppression of spontaneous activity in spastic muscles, and thus less “noise” during voluntary contraction of weakened spasticity muscles after BoNT.

This finding of unchanged motor performance of spastic muscles after BoNT injections is not trivial. This is in general agreement with the idea that spasticity is related to disordered motor control [49,50,51]. Therefore, spasticity reduction in selective group of stroke survivors is able to improve voluntary control, such as improved force initiation and relaxation [13,52], in addition to steady force production. Although it is possible that BoNT therapy could improve motor function and motor control, a recent meta-analysis further explained that the failure of function improvement after BoNT therapy is mainly due to muscle weakness [53]. In a selected group of stroke survivors, it is likely that BoNT therapy in combination with adjuvant therapy to strengthen spastic muscles may help improve motor function [54]. In this study, the same amount of BoNT was injected to biceps muscles with moderate-to-severe spasticity. The best dose of BoNT for spasticity reduction while preserving adequate muscle strength needs further investigation. We did not expect BoNT injections to a single spastic muscle would lead to a change in impairment or function of the arm. Therefore, motor impairment, such as Fugl-Meyer motor assessment or functional assessment, such as action research arm test (ARAT), were not performed. Another limitation is the small sample size in this study. Future studies with a larger sample size are needed. Assessments focusing on functional change or improvement in those subjects with voluntary muscle contraction will be appropriate in future studies. Furthermore, our findings cannot be generalized to those patients with spastic muscles of MAS 4 since they could not produce voluntary movement.

## 4. Conclusions

In summary, our results provide evidence that BoNT injections are able to reduce spasticity, while maintaining motor performance of the weakened spastic muscles in chronic stroke.

## 5. Materials and Methods

### 5.1. Participants

Ten stroke patients (51.7 ± 11.5 yrs; 5 men) with spastic hemiplegia participated in this study. Inclusion criteria for the stroke subjects were: (1) hemiplegia secondary to an ischemic or hemorrhagic stroke; (2) at least six months post-stroke; (3) residual voluntary elbow flexion force, at least be able to antigravity; (4) rated as Modified Ashworth Scale (MAS) score between 2–3; (5) elbow flexor spasticity requiring 100 units of onabotulium toxin A or incobotulinum toxin A injections to spastic biceps brachii muscle; and (6) able to understand and follow instructions related to the experiment. Exclusion criteria included stroke survivors with: (1) multiple strokes; (2) elbow flexors spasticity less than 2 or greater than 3; (3) increased resistance from other etiologies, such as rigidity and contracture; (4) a history of prior musculoskeletal injury; and (5) other comorbidities that may cause spasticity, such as spinal cord injury. The Committee for the Protection of Human Subjects at the University of Texas Health Science Center at Houston and the University of Houston approved the procedures of this study (IRB#: CPHS HSC-MS-17-0174, date of approval: 21 March 2017). All participants provided written informed consent before participating in the study. The detailed information of the stroke subjects is listed in Table 1.

### 5.2. Experimental Protocol

All subjects participated in two experimental sessions. The first session (pre-injection) was scheduled within the week before the scheduled BoNT injection, and the second session was scheduled 3~4 weeks after the injection (3-wks follow-up). The following laboratory tasks were performed by the subjects during each session: (1) maximum voluntary contraction (MVC) of elbow flexors; (2) force control; (3) passive stretch torque, in addition to clinical assessment of spasticity using Modified Ashworth Scale (MAS).

MVC was tested for both impaired and non-impaired elbow flexors, separately. Subjects performed a maximum isometric elbow flexion attempt and held the force for 3~5 s. Subjects were asked to repeat the task three times. The highest force among three attempts was considered the MVC force. Adequate rest was provided between consecutive MVC attempts.

After MVC tasks, subjects were asked to perform unilateral isometric elbow flexion tasks at 10%, 30%, and 50% of their MVC forces on each side. From the beginning of each trial, a target horizontal red line was shown on the monitor. Subjects’ force trace was shown on the monitor as a white line, running from left to right on the monitor during each 12 s trial. Subjects were asked to match their force trace (white line) to the target red line as precisely as they could. One to three practice trials were given to each subject. Three trials were collected on each side.

Only the impaired elbow joint was passively stretched in this study using an established experimental paradigm [11,55]. Subjects were asked to relax during the passive stretch tasks. The servo motor moved the forearm for 50° and then moved it back to the initial position. During the pre-injection visit, the initial position was selected as 10 degrees less than the angle when the abnormal muscle tone could be detected during a quick manual passive stretch, i.e., a catch. This angle could then be used for both pre-injection and 3-wks follow-up visits for each subject. All subjects had the initial position greater than 50° of elbow flexion, so the end position was before the full elbow extension. A ramp–hold–ramp trial of 50° passive stretch contained two seconds of motionless baseline, passive elbow extension (ramp), two seconds of hold, passive elbow flexion (ramp), and two seconds motionless rest at the initial position. There were two different stretch speeds: 5°/s and 100°/s, and the total length of a trial depended on the stretch speed. One-minute rest was given between trials. Three trials were collected for each stretch speed.

### 5.3. Experimental Set-Up

**Figure 3:**
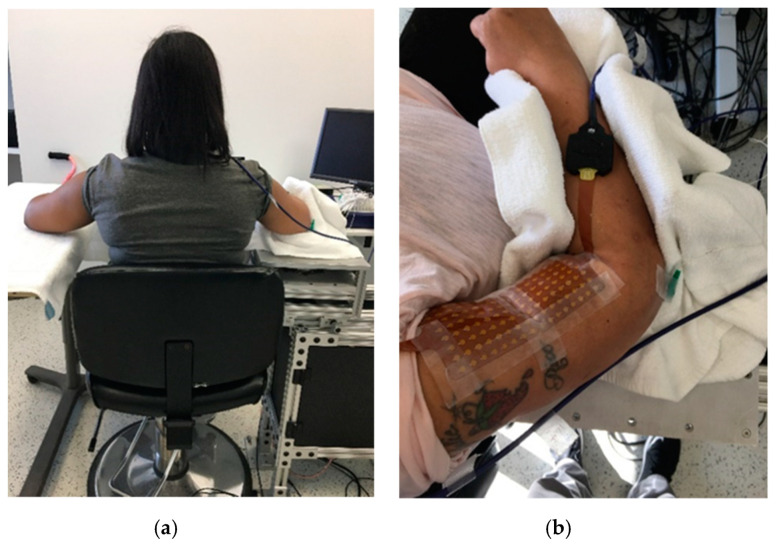
Illustration of experimental setup. (**a**) Sitting position of a representative subject with symmetrical configurations of two limbs. (**b**) The elbow angle during passive stretch task.

Subjects sat comfortably on a height adjustable chair with the targeted arm secured in a customized device in the following configuration: the shoulder joint was placed approximately in 30° of abduction and 45° of flexion, while the elbow was flexed to 90°. The wrist and fingers were kept naturally relaxed. A 20-inch computer monitor (Model: 2001FP, Dell Computer Corp., TX, USA) was put approximately one meter in front at the subject’s eye level. The monitor was used to display visual feedback of the subject’s performance using a custom-written computer program (LabView^®^, National Instrument™ Inc., Austin, TX, USA). The targeted elbow flexion force exerted during MVC, and 10% of MVC tasks was measured with a torque sensor (Model: TRS-500, Transducer Techniques, Temecula, CA, USA). The torque signal was sampled at 1000 Hz with a data acquisition card (Model: PCI-6229, National Instruments, Austin, TX, USA). In order to test the stretch reflex of the spastic elbow flexor muscle, a computerized stretching program was used to passively stretch the subject’s elbow. The axis of rotation of the elbow joint was aligned with the axis of rotation of a servomotor (model: FHA-25C-50-US250, Harmonic Drive LLC, MA, USA). Illustration of the sitting position is shown in Figure 3.

### 5.4. Data Analysis

Data collected from torque sensor was analyzed off-line using custom-written Matlab programs (MathWorksTM Inc., Natick, MA, USA). The raw torque signal was low-pass filtered at 10 Hz with a fourth-order, zero-lag Butterworth digital filter before further analysis. The following parameters were calculated:

#### 5.4.1. Force Control Performance

CV of the force produced by the subjects was used to quantify the steadiness of the force. It is a parameter used to compare the force control performance between different healthy [56,57] and patient populations [58,59]. A smaller value of CV indicates lower fluctuation of the force and better force control performance. CV of force was quantified as the standard deviation of the force divided by the mean of the force during the same period of time. In order to calculate the performance from the steadiest state during a force control task, the middle 2 s of each task was used.

#### 5.4.2. Reflex Torque

The resistance torque of a passive stretch task was calculated as the differences between the mean torque over a 200 ms window prior to stretching (the baseline) and the highest torque during the stretch as described in our recent study [11]. The resistance torque during slow stretching (5°/s) is considered as the non-reflex property of the spastic muscles (baseline torque), while the resistance torque during fast stretch (100°/s) is considered as the total resistance contained both non-reflex and reflex property of spasticity (total torque). The difference between the resistance torque of slow and fast stretches is considered as the reflex property of spasticity, reflex torque.

### 5.5. Statictical Analysis

The dependent variables in this study were: (1) MAS; (2) MVC force; (3) force control performance; (4) baseline torque; (5) total torque; (6) reflex torque. MVC and force control tasks of the non-impaired biceps muscle during 3-wks follow-up was not collected from one patient due to time restriction. Wilcoxon’s signed rank sum test was used to test the effects of injections (before and after injection) on MAS scores. A paired *t*-test was used to compare the effect of injection (before and after injection) on MVC force, baseline torque, total torque, and reflex torque. A three-way ANOVA with repeated measure were with three factors, including TIME (before and after injection), LIMBS (impaired and non-impaired), and FORCELEVEL (10%, 30%, and 50% of MVC). Pearson correlation was applied to test the correlation between reflex torque and MVC and force variability before and after BoNT injections. The alpha level for all statistical tests was 0.05. Data are reported as mean ± SD within the text and as mean ± SEM in the figures.

## Figures and Tables

**Table 1:** Characteristics of the study subjects (MAS = Modified Ashworth Scale)

ID	Age	Gender	History of Stroke (Months)	Paretic Side	Dominant Side	Elbow Flexor MAS (1st)	Elbow Flexor MAS (2nd)	Lesion Type
1	60	M	23	Left	Right	2	2	Hemorrhagic
2	52	F	104	Left	Right	2	1+	Ischemic
3	63	M	137	Left	Right	2	1+	Ischemic
4	40	F	77	Right	Right	2	1+	Ischemic
5	49	M	7	Right	Right	2	1+	Ischemic
6	40	M	39	Right	Right	2	1+	Hemorrhagic
7	68	F	7	Left	Right	2	2	Ischemic
8	65	M	29	Left	Right	2	1+	Hemorrhagic
9	39	F	76	Right	Right	2	1	Hemorrhagic
10	46	F	117	Right	Right	3	2	Ischemic
